# Hydroxyurea pharmacokinetics and precision dosing in low-resource settings

**DOI:** 10.3389/fmolb.2023.1130206

**Published:** 2023-06-01

**Authors:** Luke R. Smart, Mwesige Charles, Kathryn E. McElhinney, Min Dong, Alexandra Power-Hays, Thad Howard, Alexander A. Vinks, Emmanuela E. Ambrose, Russell E. Ware

**Affiliations:** ^1^ Division of Hematology, Cincinnati Children’s Hospital Medical Center, Cincinnati, OH, United States; ^2^ Department of Pediatrics, University of Cincinnati College of Medicine, Cincinnati, OH, United States; ^3^ Global Health Center, Cincinnati Children’s Hospital Medical Center, Cincinnati, OH, United States; ^4^ Department of Laboratory Sciences, Bugando Medical Centre, Mwanza, Tanzania; ^5^ Division of Clinical Pharmacology, Cincinnati Children’s Hospital Medical Center, Cincinnati, OH, United States; ^6^ Department of Paediatrics and Child Health, Catholic University of Health and Allied Sciences, Mwanza, Tanzania; ^7^ Department of Paediatrics and Child Health Bugando Medical Centre, Mwanza, Tanzania

**Keywords:** sickle cell disease, hydroxyurea, pharmacokinetics, precision dosing, high performance liquid chromatography

## Abstract

**Introduction:** Hydroxyurea is effective disease-modifying treatment for sickle cell anemia (SCA). Escalation to maximum tolerated dose (MTD) achieves superior benefits without additional toxicities, but requires dose adjustments with serial monitoring. Pharmacokinetic (PK)-guided dosing can predict a personalized optimal dose, which approximates MTD and requires fewer clinical visits, laboratory assessments, and dose adjustments. However, PK-guided dosing requires complex analytical techniques unavailable in low-resource settings. Simplified hydroxyurea PK analysis could optimize dosing and increase access to treatment.

**Methods:** Concentrated stock solutions of reagents for chemical detection of serum hydroxyurea using HPLC were prepared and stored at −80C. On the day of analysis, hydroxyurea was serially diluted in human serum, then spiked with N-methylurea as an internal standard and analyzed using two commercial HPLC machines: 1) standard benchtop Agilent with 449 nm detector and 5 micron C18 column; and 2) portable PolyLC with 415 nm detector and 3.5 micron C18 column. After validation in the United States, the portable HPLC and chemicals were transported to Tanzania.

**Results:** A calibration curve using hydroxyurea 2-fold dilutions ranging from 0 to 1000 µM was plotted against the hydroxyurea:N-methylurea ratio. In the United States, both HPLC systems yielded calibration curves with R^2^ > 0.99. Hydroxyurea prepared at known concentrations confirmed accuracy and precision within 10%–20% of the actual values. Both HPLC systems measured hydroxyurea with <10% variance from the prepared concentrations, and paired analysis of samples on both machines documented <15% variance. Serial measurements of 300 and 100 μM concentrations using the PolyLC system were precise with 2.5% coefficient of variance. After transport to Tanzania with setup and training, the modified PolyLC HPLC system produced similar calibration curves with R^2^ > 0.99.

**Conclusion:** Increasing access to hydroxyurea for people with SCA requires an approach that eases financial and logistical barriers while optimizing safety and benefits, especially in low-resource settings. We successfully modified a portable HPLC instrument to quantify hydroxyurea, validated its precision and accuracy, and confirmed capacity building and knowledge transfer to Tanzania. HPLC measurement of serum hydroxyurea is now feasible in low-resource settings using available laboratory infrastructure. PK-guided dosing of hydroxyurea will be tested prospectively to achieve optimal treatment responses.

## 1 Introduction

Hydroxyurea is a highly effective disease-modifying treatment for sickle cell anemia (SCA) (Ware, 2010). It is approved for treatment of both children and adults with SCA in the United States (US) and recommended for all affected children as young as 9 months of age (Yawn et al., 2014). Hydroxyurea is especially vital for low-resource settings where the vast majority of people with SCA live (Piel et al., 2013; Power-Hays and Ware, 2020) and has been designated an essential medicine by the World Health Organization (WHO, 2021a; WHO, 2021b).

Hydroxyurea treatment is safe and effective in low-resource and malaria endemic regions of the world (Opoka et al., 2017; Tshilolo et al., 2019). Access to hydroxyurea for SCA in low-resource settings is gradually expanding, but the optimal approach to dosing and monitoring is still a subject of debate. The rates of absorption, distribution, metabolism, and excretion of hydroxyurea vary widely among individuals (de Montalembert et al., 2006; Ware et al., 2011), hence neither a single fixed dose nor a single weight-based dose is appropriate for all people. Traditionally, a weight-based dose of hydroxyurea is initiated in a moderate range (10–20 mg/kg/day) to avoid toxicities, and then the hematological effects are monitored to facilitate incremental dose adjustments based on myelosuppression, until an optimal response is achieved. In low-resource settings where the cost and logistics of laboratory monitoring are onerous, alternative low-dose regimens have been suggested (Abdullahi et al., 2022a; Abdullahi et al., 2022b), but individualized escalated dosing provides superior laboratory and clinical benefits without incurring any additional toxicities (John et al., 2020).

Pharmacokinetic (PK)-guided dosing is an alternate personalized dosing strategy based on the patient’s own parameters, and we have developed this approach for hydroxyurea that features 1) a single oral test dose; 2) sparse blood sampling to model drug absorption, distribution, metabolism, and excretion; and 3) a model-informed precision dosing algorithm to estimate the drug area-under-the-curve (AUC) and to predict a personalized optimal dose (Dong et al., 2016). This methodological approach should require fewer clinical visits, laboratory assessments, and dose adjustments, and was shown in the US to shorten the time to achieve an optimal dose without increasing hematological toxicities (McGann et al., 2019). Currently, measurement of serum hydroxyurea concentrations used for PK-guided dosing requires expensive, complex analyses that are unavailable in low-resource settings (Marahatta et al., 2016).

An improved method for hydroxyurea PK analysis, coupled with a user-friendly dosing algorithm, could simplify hydroxyurea dosing and monitoring. Our laboratory has developed a method for accurate quantitative measurement of hydroxyurea concentrations in body fluids using a large benchtop high performance liquid chromatography (HPLC) instrument (Marahatta and Ware, 2017). We recently modified this method using easily sourced chemicals and supplies, to allow analysis with a smaller and portable low-cost HPLC machine with calculation of drug PK and dosing prediction. These improvements should improve access to PK-guided precision dosing and increase access to hydroxyurea treatment in low-resource settings.

## 2 Materials and methods

### 2.1 Chemicals, reagents, and stock solutions

In our US lab, hydroxyurea, iron (III) chloride, diacetylmonoxime, thiosemicarbazide, trichloracetic acid, acetonitrile (ACN), phosphoric acid, and sulfuric acid and pooled, heat-inactivated human serum were obtained from Sigma-Aldrich. N-methylurea was purchased from Santa Cruz Biotechnologies. Millipore filtered water from the lab and HPLC grade ACN were used for the mobile phases.

### 2.2 Durable equipment

A−80C freezer was used for storage of stock solutions. A high speed (13,000 rpm) microcentrifuge and heat block with 100C capacity were used for preparation of samples prior to analysis.

### 2.3 Preparation of standard solutions

Concentrated stock solutions were prepared of hydroxyurea (1 M), N-methyl-urea (4.5 mM), ferric (III) chloride (0.015 M), diacetylmonoxime (0.37 M), and thiosemicarbazide (0.06 M). Small single use aliquots were prepared with enough volume to prepare a calibration curve and 12 human specimens on 1 day of testing and stored in 1.5 mL Eppendorf tubes at −80C. Trichloroacetic acid was dissolved in water to make a 100% solution and stored at room temperature in an opaque bottle, away from exposure to direct sunlight.

On the day of hydroxyurea analysis, aliquots of the stock solutions were removed from the freezer and allowed to warm up to room temperature without heating. A coloring solution was created by adding 500 μL of 0.37 M diacetylmonoxime and 500 μL of 0.06 M thiosemicarbazide to 9 mL of water. An acid solution was created by adding 100 μL of 0.015 M ferric (III) chloride, 760 μL of 100% sulfuric acid, and 5 μL of 85% phosphoric acid to 9.135 mL of water.

**FIGURE 1:**
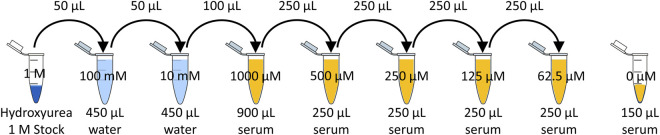
Serial dilution of hydroxyurea.

The 1 M hydroxyurea stock was serially diluted in water to create a 10 mM solution, and then serially diluted to create 5 dilutions in human serum of 1,000 µM (76.1 μg/mL), 500 µM (38.0 μg/mL), 250 µM (19.0 μg/mL), 125 µM (9.5 μg/mL), and 62.5 µM (4.8 μg/mL). Figure 1 depicts the serial dilution in water and serum to achieve the desired calibrators.

### 2.4 Calibration curve of hydroxyurea

A calibration curve was created using measurements of the 5 concentrations of hydroxyurea serially diluted in human serum (Figure 1). A 150 µL aliquot of each dilution was added to a new microcentrifuge tube. Each was spiked with 20 µL of 4.5 mM N-methylurea as an internal standard. Each specimen was deproteinated by adding 20 µL of trichloroacetic acid, vortexing, then performing centrifugation at 13,000 rpm for 15 min. Then 150 µL of supernatant were transferred to a new microcentrifuge tube, and 500 µL of both the acid and color reagent were added to each tube. These were mixed thoroughly and placed in a 100C heat block for 10 min. Then the microcentrifuge tubes were transferred to an ice bucket and covered to avoid exposure to light. Each specimen was analyzed using HPLC to identify the appropriate peaks (hydroxyurea and methylurea) for the hydroxyurea AUC calculation. The prepared concentration of hydroxyurea was then plotted against the ratio of hydroxyurea AUC divided by the Internal Standard (N-methyl urea) AUC detected by each HPLC method.

### 2.5 Chromatography

**FIGURE 2:**
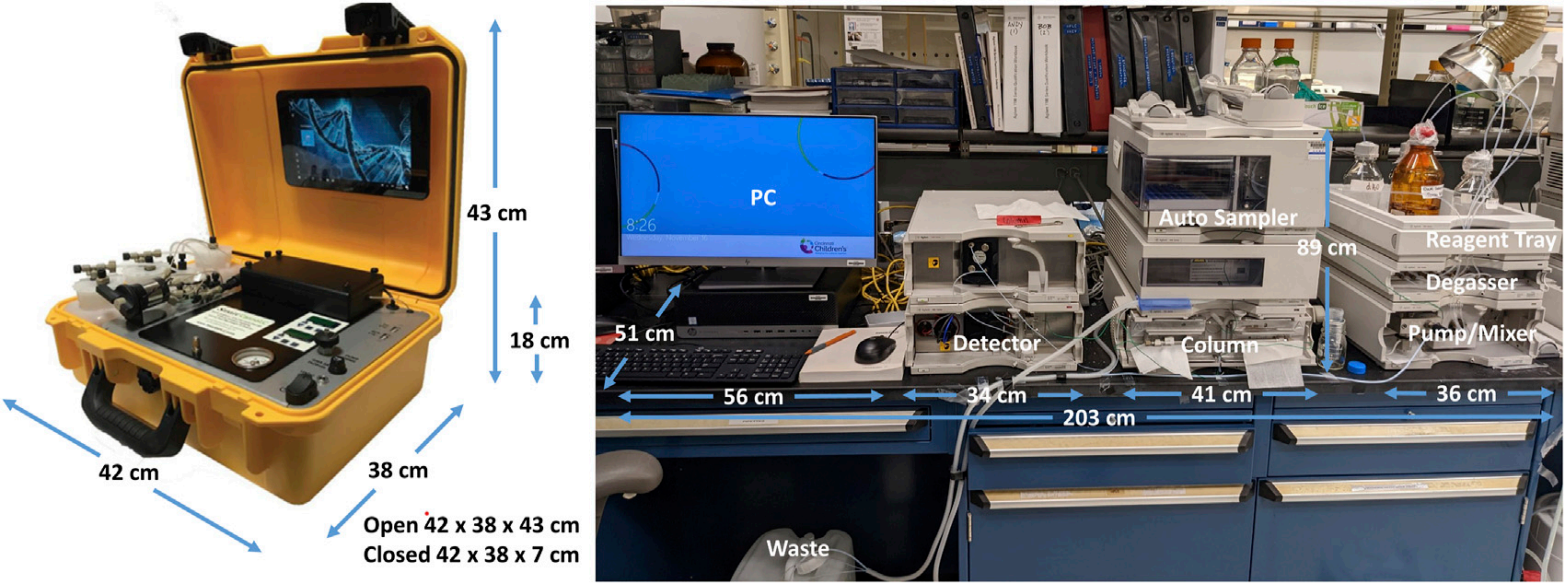
Lab footprint of Agilent (203 × 51 × 89 cm) and PolyLC HPLC Systems (42 × 38 × 43 cm).

The original HPLC method in our lab uses a benchtop Agilent (Agilent Technologies, Santa Clara, CA) instrument with a 449 nm detector and 4.6 mm × 250 mm, 5 micron Zorbax Eclipse XDB-C18 column (Agilent Technologies, Santa Clara, CA) and an automated 100 μL injection volume. The countertop footprint for this instrument is fairly large, approximately 203 cm long x 51 cm deep, plus clearance of 89 cm in height. Figure 2 displays the comparative footprints of each system. The mobile phase is 13% acetonitrile in water with a flow rate of 1 mL/min and a 15-min run time. The specimens are analyzed at 30C as described (Marahatta and Ware, 2017).

The portable HPLC suitable for low-resource settings was a PolyLC (Columbia, MD) instrument with a 415 nm detector designed for hemoglobin detection and manual injections. We modified the system by changing the injection loop from 20 μL to 100 μL and changing the column to a 4.6 mm × 50 mm, 3.5 micron Zorbax Eclipse XDB-C18 column (Agilent Technologies, Santa Clara, CA). The countertop footprint for this smaller instrument is approximately 42 cm long x 38 cm deep, plus clearance of 43 cm in height (Figure 2), and weighs approximately 10 kg.

We also modified the HPLC method to accommodate the different column and temperature of analysis, using mobile phases of acetonitrile and water as described in Table 1. Briefly, the flow rate was 1.0 mL/min and the gradient started with 10% acetonitrile for 1 min, which was then ramped up to 13% acetonitrile over 5 min and maintained for 1 min before cycling back to 10% acetonitrile over 1 min and then held for an additional 2 min before initiating the next injection. An injection volume of 100 μL was analyzed from each specimen and all analyses were performed at room temperature. The differences between instruments are summarized in Table 2.

**TABLE 1:** Adapted mobile phase method for measurement of hydroxyurea in serum using PolyLC.

Time (min)	Water (%)	Acetonitrile (%)	Flow (mL/min)
Initial	90.0	10.0	1.0
1.0	90.0	10.0	1.0
6.0	87.0	13.0	1.0
7.0	87.0	13.0	1.0
8.0	90.0	10.0	1.0
10.0	90.0	10.0	1.0

**TABLE 2:** Comparison of Agilent and adapted PolyLC for measurement of hydroxyurea in serum.

	Agilent	PolyLC
Column	4.6 mm × 250 mm, 5 micron	4.6 mm × 50 mm, 3.5 micron
Injection volume	100 μL	100 μL
Temperature Control	Yes (30°C)	No (24°C)
Mobile Phase	13% Acetonitrile	10%–13% Acetonitrile
Flow Rate	1.0 mL/min	1.0 mL/min
Injection method	Automated	Manual
Run Time	15 min	10 min
Detector Wavelength	449 nm	415 nm

### 2.6 Method validation

#### 2.6.1 Measurement of hydroxyurea at 415 nm

The pink reaction product of the Fearon reaction has maximum absorbance at 449 nm of light (Manouilov et al., 1998). The wavelength of the detector used by the Agilent HPLC system is adjustable, but the PolyLC system was initially designed to quantify hemoglobin, and it is sold with a fixed wavelength detector that is 415 nm. To determine whether hydroxyurea at lower concentrations could still be detected using 415 nm, the reaction products were measured at both 415 nm and 449 nm on the Agilent HPLC system.

#### 2.6.2 Measurement at room temperature

The PolyLC system does not have temperature control. To determine whether hydroxyurea could still be detected while the HPLC was operating at room temperature, the reaction product was measured at 24C and 415 nm on the PolyLC HPLC system and at 30C and 449 nm on the Agilent HPLC system.

#### 2.6.3 Measurement with stock solutions

Two calibration curves were created from serially diluted hydroxyurea in serum, prepared using two different sets of reagents and then analyzed using the Agilent HPLC system. One set of specimens was prepared using coloring and acid solutions that were created from fresh iron (III) chloride, diacetylmonoxime, and thiosemicarbazide. A second set of specimens were prepared using coloring and acid solutions that were created using stock solutions of 0.015 M iron (III) chloride, 0.37 M diacetylmonoxime, and 0.06 M thiosemicarbazide. The serially diluted hydroxyurea specimens from each set of specimens were both analyzed using the Agilent HPLC system.

#### 2.6.4 Stability of stock solutions

The stock solutions of iron (III) chloride, diacetylmonoxime, and thiosemicarbazide were stored at −80C. After 1 month and 1 year of storage, specimens were prepared using both fresh reagents and concentrated stock to compare the stability of reagents and the hydroxyurea concentration measured using frozen stock reagents with the concentration measured using freshly prepared reagents. The stability of hydroxyurea stored at 1 M in −80C has been previously demonstrated (Marahatta et al., 2016).

#### 2.6.5 Precision

Intra-day precision was estimated by preparing a concentrations of hydroxyurea at 300 µM (22.8 μg/mL) and 100 µM (7.6 μg/mL) in serum, preparing them for analysis similar to calibration curve specimens, and taking three serial measurements on the same day on each HPLC system. For the inter-day precision, the prepared concentrations for the calibration curve were analyzed using the same method on five consecutive days. The precision was defined as the coefficient of variance (i.e., relative standard deviation which is equal to the standard deviation divided by the mean), and it was targeted not to exceed 15% (U.S. Department of Health and Human Services Food and Drug Administration Center for Drug Evaluation and Research, 2018).

#### 2.6.6 Accuracy

Accuracy was calculated by comparing the measured hydroxyurea concentration as calculated using the HPLC to the nominal prepared concentration values. These were expressed as a percentage of the nominal value. The accuracy was considered acceptable if it was ± 15% of the nominal concentration (U.S. Department of Health and Human Services Food and Drug Administration Center for Drug Evaluation and Research, 2018).

### 2.7 Transfer of technology

The PolyLC system was transported to Tanzanian as carry-on luggage on a commercial aircraft. A small quantity of HPLC grade hydroxyurea, N-methylurea, ferric (III) chloride, diacetylmonoxime, thiosemicarbazide, and trichloroacetic acid purchased from Sigma-Aldrich were transported via checked luggage on commercial aircraft. Acetonitrile cannot be transported on commercial aircraft; therefore, a supply of HPLC grade acetonitrile was purchased from a Merck distributor in Dar es Salaam, Tanzania and shipped to the site by ground transport. Human serum was obtained from local blood donors. Distilled water was obtained from the clinical laboratory distiller.

The HPLC system was set-up in the clinical laboratory at Bugando Medical Centre, a large tertiary care and referral center in Mwanza, Tanzania. The clinical laboratory was using a centrifuge with maximum speed of 3,000 rpm for preparation of clinical specimens, so a microcentrifuge with maximum speed of 13,000 rpm was purchased and donated. The clinical laboratory used either a Bunsen burner or a hot plate to heat specimens or create a boiling water bath for heating specimens to 100C, so a small heat block was purchased and donated. The clinical laboratory used reusable freezer packs for ice. To chill specimens, a metal block from a hot plate was donated and stored in the freezer. Importation of donated equipment for non-profit hospitals has minimal cost if documentation is obtained from the Tanzanian Medicines and Medical Devices Authority. Equipment that is shipped via mail courier usually must be cleared from customs by a clearing agent and additional tax must be paid.

Two laboratory personnel in the clinical laboratory were trained to prepare stock solutions and small aliquots for storage at −80C. They were then trained how to prepare the calibration curve and specimens for analysis. Finally, they were trained on the principles of HPLC and the specific methods required for the PolyLC system and the hydroxyurea methods.

## 3 Results

### 3.1 Startup and operational costs

The portable PolyLC system along with components required for hydroxyurea measurement cost approximately $16,000 USD. The initial supply of chemicals and consumables required for creation of stock solutions, and analysis of specimens cost another $1,000 USD. Ongoing operational costs of performing the method in the future will include 4–6 h of technician time for analysis of specimens from three people.

### 3.2 Detection at 415 nm

**FIGURE 3:**
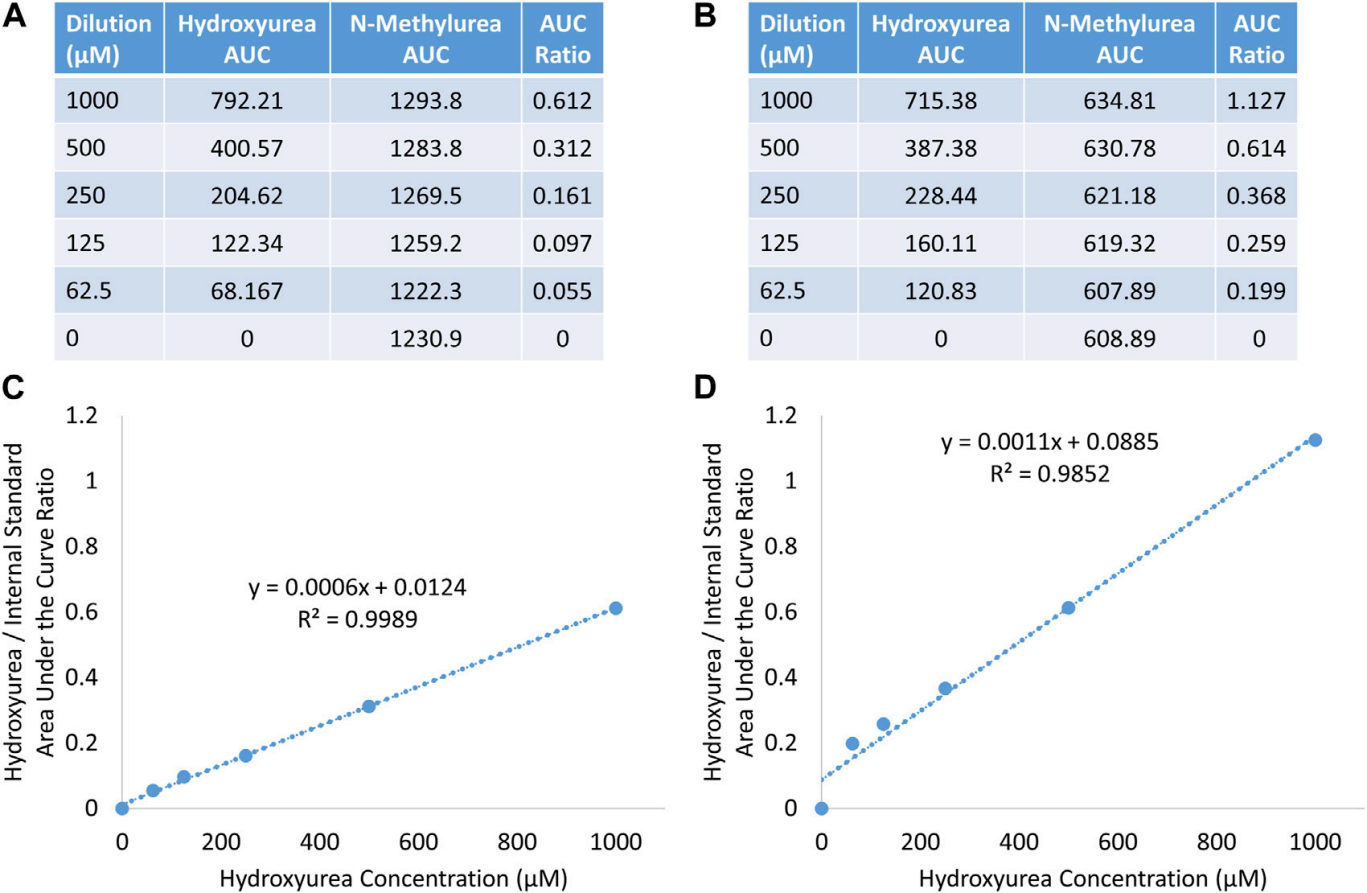
Detection of hydroxyurea and N-methylurea at 449 nm **(A, C)**, and 415 nm **(B, D)** on agilent HPLC system.

Hydroxyurea calibration curves performed at both 415 nm and 449 nm on the Agilent HPLC system documented excellent linearity with R^2^ > 0.99 (y = 0.0011x + 0.0885), indicating that the assay could be performed using the portable PolyLC HPLC system that only has a 415 nm detector. The amount of hydroxyurea internal standard detected at 415 nm was less, but a substantial amount was still detected. Figure 3, shows the AUC of hydroxyurea and N-methylurea that were detected at 449 nm (Panel A) and 415 nm (Panel B). The AUC of hydroxyurea is similar between the two. The lower wavelength detector detects half as much N-methylurea, changing the AUC ratio, but not the linearity of the calibration curve.

### 3.3 Detection at room temperature

Hydroxyurea calibration curves and quality control specimens performed on the Agilent at 30C and 449 nm compared with the PolyLC at 24C and 415 nm yielded similar results (data not shown).

### 3.4 Concentrated stock solutions

No difference in results was observed with the use of concentrated stock solutions stored at −80C compared to freshly prepared solutions. Stocks remained usable and had similar results after 1 month and 1 year of storage (data not shown).

### 3.5 Comparison of HPLC systems

#### 3.5.1 Linearity

**FIGURE 4:**
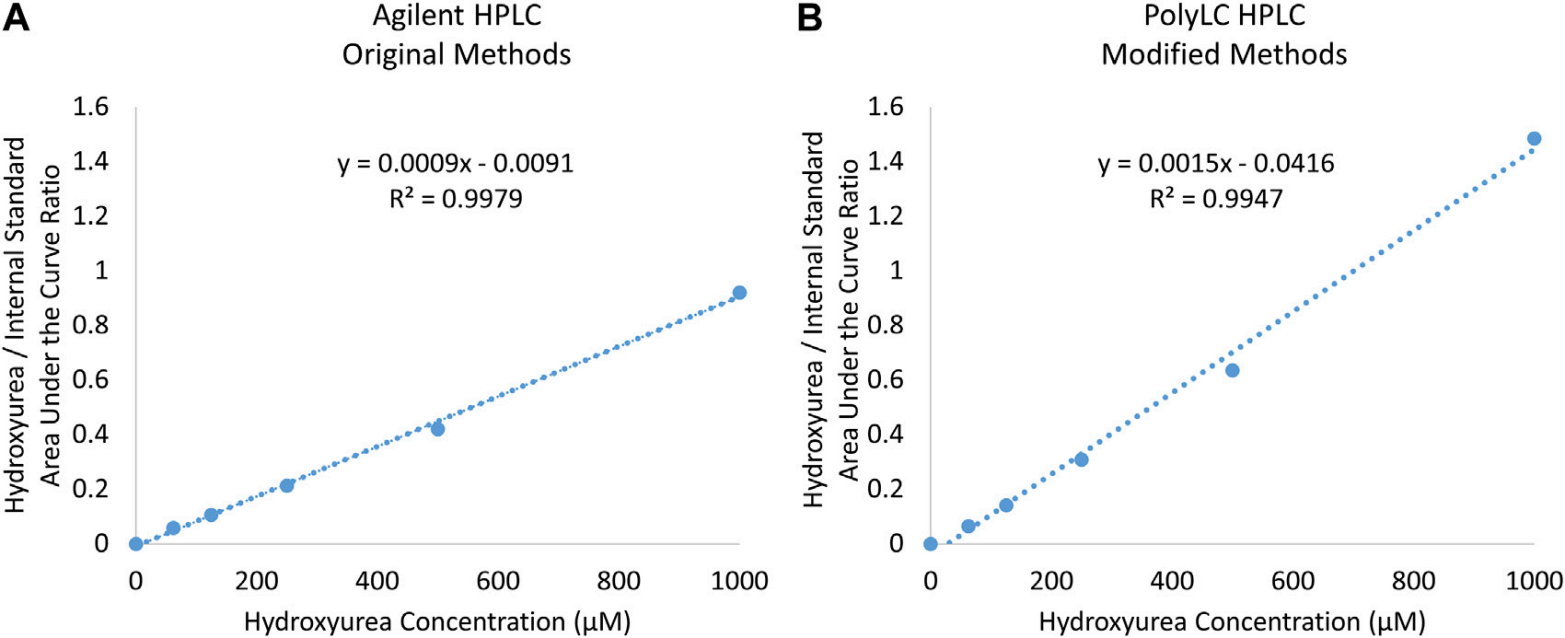
Comparison of Calibration Curves Prepared using **(A)** original Agilent and **(B)** modified PolyLC System.

In our US laboratory the results obtained on the Agilent HPLC system using original methods and the PolyLC HPLC system with modified methods were highly congruent despite the differences in detector, column length, run time, and analysis temperature. Figure 4 displays a calibration curve obtained using the original method and HPLC system side by side with modified method and system. On each machine the R^2^ exceeded 0.99. The PolyLC yielded a calibration curve a steeper slope because of the changes in N-methylurea detection described above (y = 0.0009x—0.0091 vs. y = 0.0015x—0.0416. Paired analyses of samples on both machines yield results with <15% difference (Table 3).

**TABLE 3:** Paired analysis of serially diluted calibration curve specimens on the Agilent and PolyLC.

Prepared hydroxyurea concentration (μM)	Calculated hydroxyurea concentration (μM) agilent HPLC	Calculated hydroxyurea concentration (μM) PolyLC HPLC	% Difference between machines
1,000	1,033.5	1,017.1	1.6%
500	476.9	451.0	5.4%
250	246.9	232.9	5.7%
125	127.3	121.5	4.6%
62.5	75.1	70.3	6.4%
		Mean	4.7%
		Standard Deviation	1.9

#### 3.5.2 Comparison of precision and accuracy

Three serial measurements on the same day (intra-day) of 300 µM (22.8 μg/mL) and 100 µM (7.6 μg/mL) quality control specimens analyzed using the modified chemistry were precise with a 2.5% coefficient of variance on both HPLC systems (Table 4). The results were also accurate with both systems providing results that were < 15% difference from the nominal values. Only one calibration curve was created for each machine. Serial measurements on consecutive days (inter-day) of 300 µM (22.8 μg/mL) and 100 µM (7.6 μg/mL) quality control specimens on both HPLC systems yielded similar precision (CV 1.6–5.8) and accuracy (<10% difference from the nominal values).

**TABLE 4:** Intra-day paired analysis of three serial measurements of quality control specimens of 300 µM (22.8 μg/mL) and 100 µM (7.6 μg/mL) on the Agilent and PolyLC.

Prepared Dilution (µM)	Measured concentrations (µM), Mean ± SD	Coefficient of variance (%)	Accuracy (%), Mean ± SD
300 Agilent	336.0 ± 1.1	0.3	+12.0 ± 0.4
300 PolyLC	334.5 ± 4.6	1.4	+11.5 ± 1.5
100 Agilent	108.5 ± 1.4	1.3	+8.5 ± 1.4
100 PolyLC	107.0 ± 0.3	0.3	+7.0 ± 0.3

### 3.6 Performance in Tanzania

After transport to Tanzania, setup, and training, the PolyLC system was able to produce similar excellent hydroxyurea concentration curves with R^2^ > 0.99. Precision and accuracy during 5 days of performing a calibration curve were higher than the intra-day replicates, but were still within the expected range (Table 5).

**TABLE 5:** Inter-Day Precision and Accuracy over 5 Days for serial Dilutions of 1,000–62.5 µM (76.1–4.8 μg/mL) and 2 quality control specimens of 300 µM (22.8 μg/mL) and 100 µM (7.6 μg/mL).

Prepared Dilution (µM)	Measured concentrations (µM), Mean ± SD	Coefficient of variance (%)	Inter-day accuracy (%), Mean ± SD
1,000	996.6 ± 20.1	2.0	0.3 ± 2.0
500	527 ± 35.6	6.8	5.4 ± 7.1
250	237.3 ± 14.3	6.0	−5.1 ± 5.7
125	123.9 ± 6.2	5.0	−0.9 ± 4.9
62.5	62.5 ± 11.0	17.5	0.0 ± 17.5
300	339.7 ± 32.0	9.4	13.2 ± 10.7
100	103.2 ± 11.2	10.9	3.2 ± 11.2

*the coefficient of variance is the standard deviation (SD) divided by the mean.

### 3.7 Durability and sustainability

The PolyLC system is a portable and versatile HPLC method. The system is protected in a hard plastic outer shell while not in use (Figure 2). PolyLC has successfully distributed machines to many foreign locations through mail courier. No service contract is required. One machine was transported to Tanzania via commercial air without difficulty. The chemicals used for operation can be obtained through international suppliers who have distributors in Tanzania.

## 4 Discussion

Hydroxyurea is an essential medication for children and adults with sickle cell anemia, especially in low-resources settings where it is presently the cheapest and most effective therapy available. However, widespread access and use of hydroxyurea has been hampered by a number of logistical barriers. Though it will provide some benefits at a low or moderate dose, hydroxyurea’s greatest effects are realized when the dose is tailored to the individual with incremental dose escalation to MTD (John et al., 2020). Dose escalation requires an educated and willing provider, a family capable of following instructions, and periodic laboratory evaluations to determine when an appropriate dose has been reached so that unnecessary hematological toxicities can be avoided. PK-guided dosing could shorten the time to an optimal dose and alleviate the need for prolonged dose titration and frequent monitoring of hematological parameters. PK-guided dosing requires the ability to determine serum hydroxyurea concentrations, which until now has only been accomplished with large, expensive equipement.

We have demonstrated that a modified chemical method for detection and quantification of hydroxyurea is precise, accurate, and reproducible using a low cost portable HPLC machine that has been repurposed and adjusted to accommodate measurement of serum hydroxyurea in addition to hemoglobin analysis. This new method retained excellent accuracy and precision when analyzing intraday replicates and after moving to a low-resource setting with proper training and analyzing on multiple consecutive days. The slightly higher variability in Tanzania was likely due to the fact that a new calibration curve is created for each day of use. The HPLC machine that was used for this method has been maintained on-location and operated by local personnel for analysis of hemoglobin for over 3 years. The same machine was adapted to run the hydroxyurea assay when needed. The laboratory staff can toggle back and forth between the hemoglobin assay and the hydroxyurea as needed. Maintenance and service have been accomplished through virtual teleconference. Extra supplies and parts have been delivered when personnel from collaborating programs visit the location.

As a next step we will confirm that hydroxyurea can be detected in human specimens collected and analyzed in a low-resource setting. This will require proper collection, processing, and storage of specimens prior to evaluation. We are also testing a separate hydroxyurea assay that requires less chemical manipulation and shortens the time of preparation and evaluation of specimens so that PK-guided dosing could more easily be accomplished in a busy clinical laboratory. We will be testing PK-guided precision dosing in two upcoming clinical trials in Tanzania (SPHERE, NCT 03948867) and Uganda (ADAPT, NCT 05662098) (Smart et al., 2022).

## 4.1 Conclusion

Improving access to hydroxyurea as an essential treatment for all children living with SCA requires an approach that eases financial and logistical barriers while optimizing safety and benefit, especially in low-resource settings. We have successfully modified a portable HPLC instrument to detect hydroxyurea, validated its precision and accuracy, and confirmed capacity building and knowledge transfer to a low-resource setting. Implementation of HPLC for measurement of serum hydroxyurea concentrations is now feasible in low-resource settings using available laboratory infrastructure, so treatment initiation with a personalized PK-guided optimal dose of hydroxyurea may soon be possible.

## Data Availability

The raw data supporting the conclusions of this article will be made available by the authors, without undue reservation.

## References

[B1] Abdullahi S. U. Sunusi S. M. Abba M. S. Sani S. Inuwa H. A. Gambo S. (2022a). Hydroxyurea for secondary stroke prevention in children with sickle cell anemia in Nigeria: A randomized controlled trial. Blood 141, 825–834. 10.1182/blood.2022016620 PMC10023719 36322937

[B2] Abdullahi S. U. Jibir B. W. Bello-Manga H. Gambo S. Inuwa H. Tijjani A. G. (2022b). Hydroxyurea for primary stroke prevention in children with sickle cell anaemia in Nigeria (SPRING): A double-blind, multicentre, randomised, phase 3 trial. Lancet Haematol. 9, e26–e37. 10.1016/S2352-3026(21)00368-9 34971579 PMC10072240

[B3] de Montalembert M. Bachir D. Hulin A. Gimeno L. Mogenet A. Bresson J. L. (2006). Pharmacokinetics of hydroxyurea 1,000 mg coated breakable tablets and 500 mg capsules in pediatric and adult patients with sickle cell disease. Haematologica 91, 1685–1688. 10.3324/%x 17145606

[B4] Dong M. McGann P. T. Mizuno T. Ware R. E. Vinks A. A. (2016). Development of a pharmacokinetic-guided dose individualization strategy for hydroxyurea treatment in children with sickle cell anaemia. Br. J. Clin. Pharmacol. 81, 742–752. 10.1111/bcp.12851 26615061 PMC4799926

[B5] John C. C. Opoka R. O. Latham T. S. Hume H. A. Nabaggala C. Kasirye P. (2020). Hydroxyurea dose escalation for sickle cell anemia in sub-Saharan Africa. N. Engl. J. Med. 382, 2524–2533. 10.1056/NEJMoa2000146 32579813

[B6] Manouilov K. K. McGuire T. R. Gwilt P. R. (1998). Colorimetric determination of hydroxyurea in human serum using high-performance liquid chromatography. J. Chromatogr. B Biomed. Sci. Appl. 708, 321–324. 10.1016/s0378-4347(97)00634-8 9653981

[B7] Marahatta A. Megaraj V. McGann P. T. Ware R. E. Setchell K. D. (2016). Stable-isotope dilution HPLC-electrospray ionization tandem mass spectrometry method for quantifying hydroxyurea in dried blood samples. Clin. Chem. 62, 1593–1601. 10.1373/clinchem.2016.263715 27694393 PMC6598440

[B8] Marahatta A. Ware R. E. (2017). Hydroxyurea: Analytical techniques and quantitative analysis. Blood Cells Mol. Dis. 67, 135–142. 10.1016/j.bcmd.2017.08.009 28847416

[B9] McGann P. T. Niss O. Dong M. Marahatta A. Howard T. A. Mizuno T. (2019). Robust clinical and laboratory response to hydroxyurea using pharmacokinetically guided dosing for young children with sickle cell anemia. Am. J. Hematol. 94, 871–879. 10.1002/ajh.25510 31106898 PMC6639795

[B10] Opoka R. O. Ndugwa C. M. Latham T. S. Lane A. Hume H. A. Kasirye P. (2017). Novel use of hydroxyurea in an african region with malaria (NOHARM): A trial for children with sickle cell anemia. Blood 130, 2585–2593. 10.1182/blood-2017-06-788935 29051184

[B11] Piel F. B. Patil A. P. Howes R. E. Nyangiri O. A. Gething P. W. Dewi M. (2013). Global epidemiology of sickle haemoglobin in neonates: A contemporary geostatistical model-based map and population estimates. Lancet 381, 142–151. 10.1016/S0140-6736(12)61229-X 23103089 PMC3547249

[B12] Power-Hays A. Ware R. E. (2020). Effective use of hydroxyurea for sickle cell anemia in low-resource countries. Curr. Opin. Hematol. 27, 172–180. 10.1097/MOH.0000000000000582 32205588 PMC9364839

[B13] Smart L. R. Ambrose E. E. Balyorugulu G. Songoro P. Shabani I. Komba P. (2022). Stroke prevention with hydroxyurea enabled through research and education: A phase 2 primary stroke prevention trial in sub-saharan africa. Acta Haematol. 146, 95–105. 10.1159/000526322 35977532 PMC10100573

[B14] Tshilolo L. Tomlinson G. Williams T. N. Santos B. Olupot-Olupot P. Lane A. (2019). Hydroxyurea for children with sickle cell anemia in sub-Saharan Africa. N. Engl. J. Med. 380, 121–131. 10.1056/NEJMoa1813598 30501550 PMC6454575

[B15] U.S. Department of Health and Human Services Food and Drug Administration Center for Drug Evaluation and Research (2018). Bioanalytical method validation guidance for industry. Available at: https://www.fda.gov/media/70858/download (Accessed May 24, 2018).

[B16] Ware R. E. (2010). How I use hydroxyurea to treat young patients with sickle cell anemia. Blood 115, 5300–5311. 10.1182/blood-2009-04-146852 20223921 PMC2902131

[B17] Ware R. E. Despotovic J. M. Mortier N. A. Flanagan J. M. He J. Smeltzer M. P. (2011). Pharmacokinetics, pharmacodynamics, and pharmacogenetics of hydroxyurea treatment for children with sickle cell anemia. Blood 118, 4985–4991. 10.1182/blood-2011-07-364190 21876119 PMC3208303

[B18] WHO (2021b). World Health organization model list of essential medicines for children – 8th list, 2021. Geneva: World Health Organization.

[B19] WHO (2021a). World Health organization model list of essential medicines – 22nd list, 2021. Geneva: World Health Organization.

[B20] Yawn B. P. Buchanan G. R. Afenyi-Annan A. N. Ballas S. K. Hassell K. L. James A. H. (2014). Management of sickle cell disease: Summary of the 2014 evidence-based report by expert panel members. JAMA 312, 1033–1048. 10.1001/jama.2014.10517 25203083

